# Breakthroughs in Medicinal Chemistry: New Targets and Mechanisms, New Drugs, New Hopes–7

**DOI:** 10.3390/molecules25132968

**Published:** 2020-06-28

**Authors:** Michael Gütschow, Jean Jacques Vanden Eynde, Josef Jampilek, CongBao Kang, Arduino A. Mangoni, Paola Fossa, Rafik Karaman, Andrea Trabocchi, Peter J. H. Scott, Jóhannes Reynisson, Simona Rapposelli, Stefania Galdiero, Jean-Yves Winum, Chiara Brullo, Katalin Prokai-Tatrai, Arun K. Sharma, Matthieu Schapira, Yasu-Taka Azuma, Laura Cerchia, Mariana Spetea, Giangiacomo Torri, Simona Collina, Athina Geronikaki, Alfonso T. García-Sosa, M. Helena Vasconcelos, Maria Emília Sousa, Ivan Kosalec, Tiziano Tuccinardi, Iola F. Duarte, Jorge A. R. Salvador, Massimo Bertinaria, Maurizio Pellecchia, Jussara Amato, Giulio Rastelli, Paula A. C. Gomes, Rita C. Guedes, Jean-Marc Sabatier, Ana Estévez-Braun, Bruno Pagano, Stefano Mangani, Rino Ragno, George Kokotos, Margherita Brindisi, Florenci V. González, Fernanda Borges, Mariarosaria Miloso, Jarkko Rautio, Diego Muñoz-Torrero

**Affiliations:** 1 Pharmaceutical Institute, Pharmaceutical & Medicinal Chemistry, University of Bonn, An der Immenburg 4, 53121 Bonn, Germany; guetschow@uni-bonn.de; 2 Formerly head of the Department of Organic Chemistry (FS), University of Mons-UMONS, 7000 Mons, Belgium; jean-jacques.vandeneynde@ex.umons.ac.be; 3 Department of Analytical Chemistry, Faculty of Natural Sciences, Comenius University, Ilkovicova 6, 842 15 Bratislava, Slovakia; josef.jampilek@gmail.com; 4 Experimental Drug Development Centre, Agency for Science, Technology and Research, 10 Biopolis Road, Chromos, 05-01, Singapore 138670, Singapore; cbkang@eddc.a-star.edu.sg; 5 Discipline of Clinical Pharmacology, College of Medicine and Public Health, Flinders University and Flinders Medical Centre, Bedford Park, Adelaide 5042, Australia; arduino.mangoni@flinders.edu.au; 6 Medizinische Fakultät Carl Gustav Carus, Technische Universität Dresden, 01069 Dresden, Germany; 7 Department of Pharmacy, School of Medical and Pharmaceutical Sciences, University of Genova, 16132 Genova, Italy; fossa@difar.unige.it; 8 Pharmaceutical & Medicinal Chemistry Department, Faculty of Pharmacy, Al-Quds University, Jerusalem P.O. Box 20002, Palestine; dr_karaman@yahoo.com; 9 Department of Sciences, University of Basilicata, Viadell’Ateneo Lucano 10, 85100 Potenza, Italy; 10 Department of Chemistry “Ugo Schiff”, University of Florence, via della Lastruccia 13, I-50019 Sesto Fiorentino, Florence, Italy; andrea.trabocchi@unifi.it; 11 Department of Radiology, University of Michigan, Ann Arbor, MI 48109, USA; pjhscott@med.umich.edu; 12 School of Pharmacy and Bioengineering, Keele University, Keele, Staffordshire ST5 5BG, UK; j.reynisson@keele.ac.uk; 13 Laboratory of Medicinal Chemistry, Department of Pharmacy, University of Pisa, 56126 Pisa, Italy; simona.rapposelli@unipi.it; 14 Interdepartmental Research Centre of Ageing Biology and Pathology, University of Pisa, 56126 Pisa, Italy; 15 Department of Pharmacy, University of Naples Federico II, Via Mezzocannone 16, 80134 Naples, Italy; stefania.galdiero@unina.it (S.G.); jussara.amato@unina.it (J.A.); bruno.pagano@unina.it (B.P.); margherita.brindisi@unina.it (M.B.); 16 Institut des Biomolécules Max Mousseron (IBMM) UMR 5247 CNRS, ENSCM, Université de Montpellier, CEDEX 05, 34296 Montpellier, France; jean-yves.winum@umontpellier.fr; 17 Department of Pharmacy, Section of Medicinal Chemistry, University of Genoa, V.le Benedetto XV 3, I-16132 Genova, Italy; brullo@difar.unige.it; 18 Department of Pharmacology and Neuroscience, University of North Texas Health Science Center, 3500 Camp Bowie Blvd, Fort Worth, TX 76107, USA; Katalin.Prokai@unthsc.edu; 19 Department of Pharmacology, Penn State Cancer Institute, CH72, Penn State College of Medicine, 500 University Drive, Hershey, PA 17033, USA; asharma1@pennstatehealth.psu.edu; 20 Structural Genomics Consortium, University of Toronto, MaRS Centre, South Tower, 101 College St., Suite 700, Toronto, ON M5G 1L7, Canada; matthieu.schapira@utoronto.ca; 21 Department of Pharmacology and Toxicology, University of Toronto, 1 King’s College Circle, Toronto, ON M5S 1A8, Canada; 22 Laboratory of Veterinary Pharmacology, Division of Veterinary Science, Osaka Prefecture University Graduate School of Life and Environmental Sciences, 1-58 Rinku-ohraikita, Izumisano, Osaka 598-8531, Japan; azuma@vet.osakafu-u.ac.jp; 23 Institute of Experimental Endocrinology and Oncology “G. Salvatore” (IEOS), National Research Council (CNR), 80131 Naples, Italy; cerchia@unina.it; 24 Department of Pharmaceutical Chemistry, Institute of Pharmacy and Center for Molecular Biosciences Innsbruck (CMBI), University of Innsbruck, 6020 Innsbruck, Austria; mariana.spetea@uibk.ac.at; 25 Istituto di Ricerche Chimiche e Biochimiche “G. Ronzoni”, via Giuseppe Colombo 81, 20133 Milano, Italy; torri@ronzoni.it; 26 Department of Drug Sciences, Medicinal Chemistry and Pharmaceutical Technology Section, University of Pavia, Viale Taramelli 12, 27100 Pavia, Italy; simona.collina@unipv.it; 27 Department of Pharmaceutical Chemistry, School of Pharmacy, Faculty of Health Sciences, Aristotle University of Thessaloniki, 54124 Thessaloniki, Greece; geronik@pharm.auth.gr; 28 Institute of Chemistry, University of Tartu, Ravila 14a, 50411 Tartu, Estonia; alfonso.tlatoani.garcia.sosa@ut.ee; 29 i3S-Instituto de Investigação e Inovação em Saúde, Universidade do Porto, Rua Alfredo Allen 208, 4200-135 Porto, Portugal; hvasconcelos@ipatimup.pt; 30 Cancer Drug Resistance Group-IPATIMUP-Institute of Molecular Pathology and Immunology of the University of Porto, Rua Júlio Amaral de Carvalho, 45, 4200-135 Porto, Portugal; 31 Department of Biological Sciences, FFUP-Faculty of Pharmacy, University of Porto, Rua de Jorge Viterbo Ferreira 228, 4050-313 Porto, Portugal; 32 Laboratório de Química Orgânica e Farmacêutica, Departamento de Ciências, Químicas, Faculdade de Farmácia, Universidade do Porto, Rua Jorge Viterbo Ferreira 228, 4050-313 Porto, Portugal; esousa@ff.up.pt; 33 Interdisciplinar de Investigação Marinha e Ambiental (CIIMAR/CIMAR), Universidade do Porto, Terminal de Cruzeiros do Porto de Leixões, Avenida General Norton de Matos, S/N 4450-208 Matosinhos, Portugal; 34 Faculty of Pharmacy and Biochemistry, University of Zagreb, A. Kovačića 1, HR-10000 Zagreb, Croatia; ikosalec@pharma.hr; 35 Department of Pharmacy, University of Pisa, Via Bonanno 6, 56126 Pisa, Italy; tiziano.tuccinardi@unipi.it; 36 Department of Chemistry, CICECO—Aveiro Institute of Materials, University of Aveiro, 3810-193 Aveiro, Portugal; ioladuarte@ua.pt; 37 Laboratory of Pharmaceutical Chemistry, Faculty of Pharmacy, University of Coimbra, 3000-548 Coimbra, Portugal; salvador@ci.uc.pt; 38 Dipartimento di Scienza e Tecnologia del Farmaco, Università degli Studi di Torino, Via P. Giuria 9, 10125 Torino, Italy; massimo.bertinaria@unito.it; 39 Division of Biomedical Sciences, School of Medicine, University of California Riverside, Riverside, CA 92521, USA; Maurizio.Pellecchia@medsch.ucr.edu; 40 Department of Life Sciences, University of Modena and Reggio Emilia, Via Giuseppe Campi 103, 41125 Modena, Italy; giulio.rastelli@unimore.it; 41 LAQV-REQUIMTE, Departamento de Química e Bioquímica, Faculdade de Ciências da Universidade do Porto, Rua do Campo Alegre 687, 4169-007 Porto, Portugal; pgomes@fc.up.pt; 42 iMed.Ulisboa and Faculdade de Farmácia, Universidade de Lisboa, 1649-003 Lisbon, Portugal; rguedes@ff.ulisboa.pt; 43 Institute of NeuroPhysiopathology, UMR 7051, Faculté de Médecine Secteur Nord, 51, Boulevard Pierre Dramard-CS80011, CEDEX 15, 13344-Marseille, France; sabatier.jm1@libertysurf.fr; 44 Departamento de Química Orgánica, Instituto Universitario de Bio-Orgánica (CIBICAN), Universidad de La Laguna, 38206 Tenerife, Spain; aestebra@ull.edu.es; 45 Department of Biotechnology, Chemistry and Pharmacy, DoE 2018-2022, University of Siena, via Aldo Moro 2, 53100 Siena, Italy; stefano.mangani@unisi.it; 46 Department of Drug Chemistry and Technology, Rome Center for Molecular Design, Sapienza University, P.le Aldo Moro 5, 00185 Rome, Italy; rino.ragno@uniroma1.it; 47 Department of Chemistry, National and Kapodistrian University of Athens, Panepistimiopolis, 15771 Athens, Greece; gkokotos@chem.uoa.gr; 48 Departament de Química Inorgànica i Orgànica, Universitat Jaume I, 12080 Castelló, Spain; fgonzale@uji.es; 49 CIQUP/Department of Chemistry and Biochemistry, Faculty of Sciences, University of Porto, R. Campo Alegre 1021/1055, 4169-007 Porto, Portugal; fborges@fc.up.pt; 50 School of Medicine and Surgery, Experimental Neurology Unit, University of Milano-Bicocca, Via Cadore 48, 20900 Monza, MB, Italy; mariarosaria.miloso@unimib.it; 51 School of Pharmacy, Faculty of Health Sciences, University of Eastern Finland, P.O. Box 1627, FI-70211 Kuopio, Finland; jarkko.t.rautio@uef.fi; 52 Laboratory of Medicinal Chemistry, Faculty of Pharmacy and Food Sciences, and Institute of Biomedicine (IBUB), University of Barcelona, Av. Joan XXIII, 27-31, E-08028 Barcelona, Spain

## 1. Introduction

Breakthroughs in Medicinal Chemistry: New Targets and Mechanisms, New Drugs, New Hopes is a series of editorials which is published on a biannual basis by the Editorial Board of the Medicinal Chemistry section of the journal *Molecules*. In these editorials, we highlight in brief reports (of about one hundred words) a number of recently published articles that describe crucial findings, such as the discovery of novel drug targets and mechanisms of action or novel classes of drugs, which may inspire future medicinal chemistry endeavors devoted to addressing prime unmet medical needs.

## 2. Targeting MAO Isoforms by *cis*/*trans* Isomers

Highlighted by Michael Gütschow

Human monoamine oxidases (hMAO) A and B, two therapeutically relevant isoforms for central nervous disorders, share 70% sequence identity and are distinguished by different substrate specificities and inhibitor sensitivities. The groups of Stanislav Gobec (Faculty of Pharmacy, University of Ljubljana, Slovenia), Mariel Marder (Facultad de Farmacia y Bioquimica, Universidad de Buenos Aires, Argentina) and Claudia Binder (Department of Biology and Biotechnology, University of Pavia, Italy) have discovered the first example of hMAO-A and hMAO-B inhibitor pairs (1-propargyl-4-styrylpiperidines) whose selectivity is conferred by the configurational *cis*/*trans* isomerism [1]. Thus, the authors provided an impressive example of *cis* and *trans* isomers with different pharmacological activities resulting from a discrimination between structurally related enzyme isoforms. An expanded library of test compounds synthesized, and enzyme inhibition was investigated by kinetic analysis, UV/VIS measurements and X-ray crystallography and confirmed ex vivo in tissue homogenates as well as in vivo in mice. The results of this study are definitely useful for the design of further selective inhibitors of the two MAO isoforms.

## 3. A New Lead in the Combat Against the Multi-Drug Resistant *Mycobacterium Abscessus*

Highlighted by Jean Jacques Vanden Eynde

**Figure 1:**
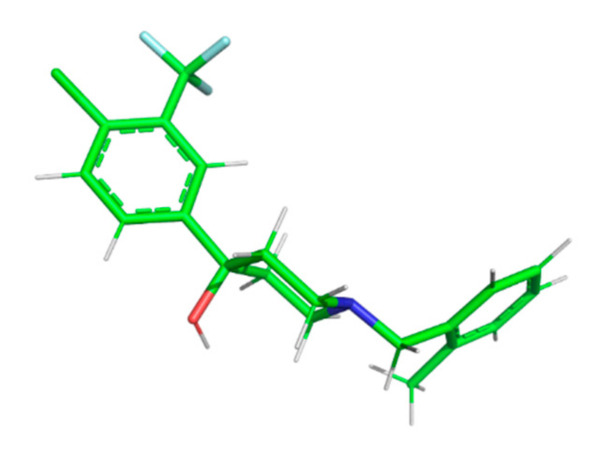
Representative example (PIPD1) of a piperidinol-containing molecule evaluated by de Ruyck et al. [2].

*Mycobacterium abscessus* is a nontuberculous ubiquitous pathogen responsible for different kinds of opportunistic infections, including pulmonary attacks and skin and soft tissues diseases. The species is resistant to most antibiotics and disinfectants, and many treatments require the use of an association of multiple drugs. In a recent study, de Ruyck et al. [2] focused their attention on the design, synthesis, and evaluation of piperidinol-containing molecules (Figure 1) that target the flippase activity of the mycolic acid transporter MmpL3. They have demonstrated that such an inhibition can result directly in the death of the pathogen. It is noteworthy that it could also be exploited in order to create synergetic effects by facilitating penetration of other substances (*i.a.* β-lactams) through the wall of the mycobacterium.

## 4. New InhA Inhibitors Provided by Fragment-Based Drug Design

Highlighted by Josef Jampilek and CongBao Kang

Fragment-based drug design (FBDD), despite its youth, has provided marketed drugs and others in clinical trials. It has become an effective strategy for designing lead compounds [3]. The library used in FBDD contains several thousand fragments and is still growing [4]. FBDD usually includes three steps: fragment screening, confirmation and growth. A recent study by Sabbah et al. has shown the application of FBDD to the design of original inhibitors of *Mycobacterium tuberculosis* InhA, which is an important target for tuberculosis. Differential scanning fluorimetry was used to screen a library of 800 fragments, and 18 hits were identified. NMR and X-ray methods were used to confirm the identified hits. The FBDD approach led from 3-trifluoromethylcinnamic acid to potent InhA inhibitors based on the *N*-[3-(aminomethyl)phenyl]-1-benzothiophene-2-sulfonamide scaffold with submicromolar IC_50_ values [5]. Although the real minimum inhibitory/bactericidal concentrations, the potential of the compounds to generate resistant/cross-resistant strains, the stability, and the ADME properties of the compounds should be investigated, this study demonstrated that FBDD is helpful for developing new inhibitors of a well-studied enzyme using a routine strategy, while careful and rational chemical design is required.

## 5. Combined Modulation of Farnesoid X Receptor and Target Genes: A Promising Therapeutic Strategy in Non-Alcoholic Steatohepatitis

Highlighted by Arduino A. Mangoni

Novel, highly effective therapeutic strategies are urgently needed for the management of non-alcoholic steatohepatitis (NASH), an increasing cause of chronic liver failure worldwide [6]. Chianelli et al. report the synthesis of a series of non-bile acid farnesoid X receptor (FXR) agonists based on a tricyclic dihydrochromenopyrazole core [7]. In particular, compound **1**, 4-[(*N*-benzyl-8-chloro-1-methyl-1,4-dihydrochromeno[4,3-*c*]pyrazole-3-carboxamido)methyl]benzoic acid exhibited partial FXR agonistic activity in vitro and FXR-dependent gene modulation in vivo. These effects were associated with a favorable pharmacokinetic profile in rodents, particularly, high bioavailability, moderate volume of distribution and clearance, and short half-life. Furthermore, in an insulin-deficient murine NASH model, treatment with compound **1** caused a significant dose-dependent reduction in parameters of liver steatosis, inflammation and fibrosis. After the successful completion of Phase 1 studies in healthy volunteers, compound **1** is currently being investigated in Phase 2 studies in patients with NASH. Pending the results of such studies, the available evidence suggests that the combined modulation of the farnesoid X receptor and target genes might represent a promising therapeutic strategy for NASH.

## 6. Cyclodextrins as a Potent Tool for Fighting Multiple Viral Infections

Highlighted by Paola Fossa and Rafik Karaman

**Figure 2:**
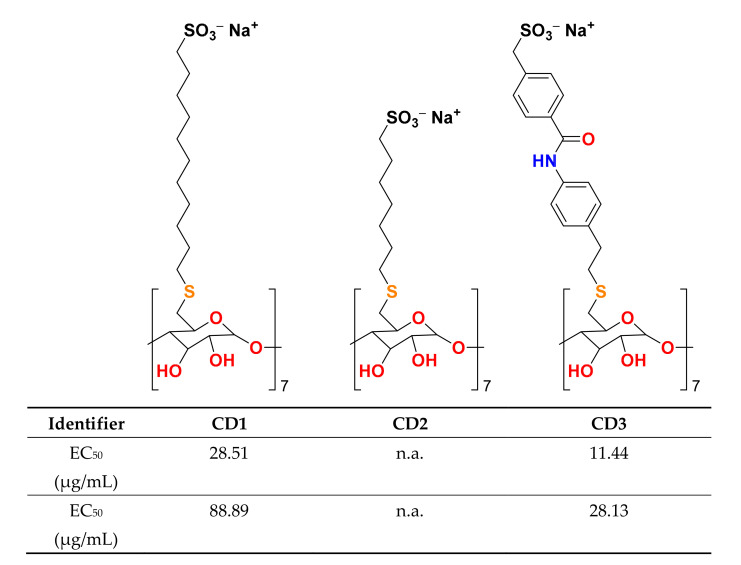
Structure of the modified cyclodextrins.

Viral infections kill millions of people and, especially now, the need for new antivirals is great. Unfortunately, all virucidal molecules described to date are strongly cytotoxic, limiting their therapeutic application in several patient types or determining severe side effects. Cyclodextrins (CD) are naturally occurring glucose derivatives with a rigid cyclic structure, consisting of alpha (1–4)-linked glucopyranoside units. Recent work by Stellacci and Tapparel [8] showed that the team successfully engineered novel modified entities utilizing cyclodextrins. These entities attract viruses before breaking them down on contact, thus causing destruction of the virus and combating the infection. The novel entities are CDs with a sulfonated alkyl chain (Figure 2), representing a new class of non-toxic, broad-spectrum, biocompatible and virucidal drugs.

This new development has the potential to derive effective treatments of herpes simplex, hepatitis C, HIV, respiratory syncytial virus, and Zika virus. Undoubtedly, the modified CDs may represent a very interesting therapeutic opportunity to be fully investigated.

## 7. Probing Amyloid Oligomers is Key to Solving the Neurodegeneration Puzzle

Highlighted by Andrea Trabocchi and Peter J. H. Scott

Many neurodegenerative disorders (NDs) are characterized by misfolding, aggregation, and accumulation of amyloid proteins. Accumulating amyloid deposits cause loss of cellular function and synaptic connections, brain damage and eventual death. Reflecting this, there has been significant interest in using amyloid deposits as an imaging biomarker to improve our understanding of mechanisms underpinning NDs [9]. Thioflavin T (ThT) is a fluorescent dye that has been used extensively to study amyloid fibril formation and was also the inspiration for development of the prototypical amyloid PET radiotracer [^11^C]Pittsburgh Compound B.

**Figure 3:**
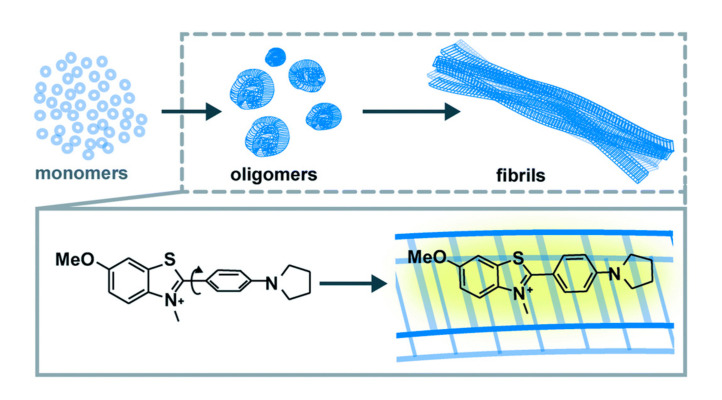
Pathway of amyloid fibril formation starting from monomeric proteins and the common cross-β sheet motif of amyloid fibrils to which benzothiazole salts bind. The binding of ThX to the fibrils restricts the rotation around the C—C bond resulting in a fluorescence turn-on response. Reprinted from Ref [11]—published by the Royal Society of Chemistry.

However, recent studies suggest that the small soluble oligomers arising during fibril formation are actually the toxic and pathogenic form of the misfolded protein, rather than the deposits themselves [10]. Unfortunately, poor photophysical and binding properties of ThT limit its use to study oligomers, and next-generation probes enabling the detection of amyloid oligomers are urgently needed. To address this, Needham et al. have developed Thioflavin X (ThX, Figure 3) [11]. ThX displayed 5- and 7-fold increases in brightness and affinity, respectively, resulting in enhanced detection of structural differences in early oligomeric species not observed via traditional ThT imaging.

## 8. Tyrosyl-DNA Phosphodiesterase 2 as a Novel Target to Enhance the Efficacy of Topoisomerase II Poisons

Highlighted by Jóhannes Reynisson

**Figure 4:**
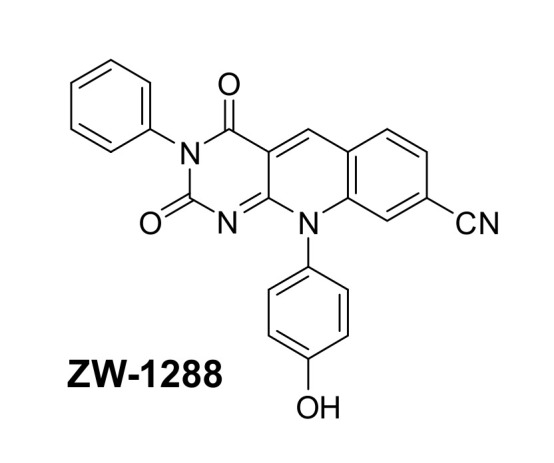
The molecular structure of ZW-1288.

The mechanism of action of many anticancer drugs in clinical use is based on damaging DNA due to the rapid growth of tumor cells. These include topoisomerase II (TOP2) poisons such as etoposide and mitoxantrone, which trap the TOP2-DNA cleavage complexes, resulting in apoptosis of the cancer cells. However, the repair enzyme Tyrosyl-DNA phosphodiesterase 2 (TDP2) can undo the trapped TOP2-DNA complexes, thus counteracting the effect of the TOP2 poisons, allowing the cancer cells to survive and develop resistance to chemotherapy. Recently, a report on an improved TDP2 inhibitor ZW-1288 (Figure 4) with low nanomolar activity was released [12]. This ligand is a deazaflavin derivative and potentiates etoposide and mitoxantrone in prostate, leukemia and lymphoma cancer cells, whilst expressing weak cytotoxicity. This demonstrates that inhibiting TDP2 with a drug-like small molecule is possible making this enzyme a very promising therapeutic target [12].

## 9. Proteostasis Network: A New Paradigm for Drug Discovery?

Highlighted by Simona Rapposelli

Oxidative stress is frequently addressed as the main cause of aging. However, different processes have been identified that contribute significantly to the onset of neurodegenerative diseases (NDDs), such as the progressive inefficiency of the proteostasis network [13]. Indeed, among the dysfunctional mechanisms occurring with aging, two protein degradation systems, the ubiquitin–proteasome system (UPP) and the autophagy–lysosome pathway (ALP) are being impaired. Therefore, the development of innovative and multivalent therapies aimed at sustaining proteome balance is a challenging strategy to identify drug treatments for chronic and disabling diseases or for aging prevention. To date, natural compounds have been recognized as multifunctional molecules capable of influencing the stability of the proteome [14], thus representing a valid starting point to design and synthesize new modulators of the proteostasis network.

## 10. The Fascinating Perspective of Material Science: Precise Embolization in Tumor Vessels Through Peptide-Based Nanoparticles

Highlighted by Stefania Galdiero

The development of cancer therapeutic strategies based on angiogenesis attracts great interest in biomedicine and biotechnology research and could lead to novel materials having great potential in clinics. Zhang et al. [15] prepared an embolus inside a tumor’s blood vessels to block them, through mimicking laminin fibrillogenesis, specifically and highly efficiently. This strategy resulted in the blockage of the tumor vessels and in a higher inhibition of tumors. In particular, the development of dual-responsive peptide-based nanoparticles made of a fibrillation sequence, a pH-responsive sequence and a targeting sequence is reported. The peptide nanoformulations are delivered to blood vessels in the tumors where the microenvironment enables formation of the fibrous network, which captures the red blood cells forming occlusions specifically in the tumor blood vessels and inhibiting the growth of the tumor. A biomimetic and promising approach to attack tumor cells and cause tumor cell death was developed, which suggests that material science may be exploited for in-situ construction of therapeutic agents for disease treatment.

## 11. Promising Anti-Leishmania Compounds in the Telluride Sulfonamide Series

Highlighted by Jean-Yves Winum

Leishmaniasis is a neglected parasitic infection that causes skin or visceral lesions and can be fatal if left untreated. Due to the emergence of resistant parasites, there is an urgent need to find new targets and to develop more effective and less toxic drugs. *Leishmania donovani chagasi* β-carbonic anhydrase (LdcCA) is playing a key role in the growth and virulence of the parasite; thus, specific LdcCA inhibitors could help fighting this disease more effectively. [16] Angeli and colleagues have reported the discovery of substituted aryl-benzyl telluride compounds, bearing a sulfonamide moiety. These compounds displayed nanomolar inhibitory activity against LdcCA. One of the best inhibitors showed high antileishmanial activity, displaying (i) a selectivity index over 300-fold higher for the *L. infantum* axenic amastigotes over THP1 cells and (ii) a selectivity up to 50 times higher than edelfosine against infected macrophages. In-vitro and in-vivo toxicity experiments have highlighted the innocuity of this compound. [17] This study provides new directions for the design and subsequent development of drug candidates targeting the carbonic anhydrase of *Leishmania* or more generally of other protozoan parasites.

## 12. New Insight in Fak Inhibitors

Highlighted by Chiara Brullo

**Figure 5:**
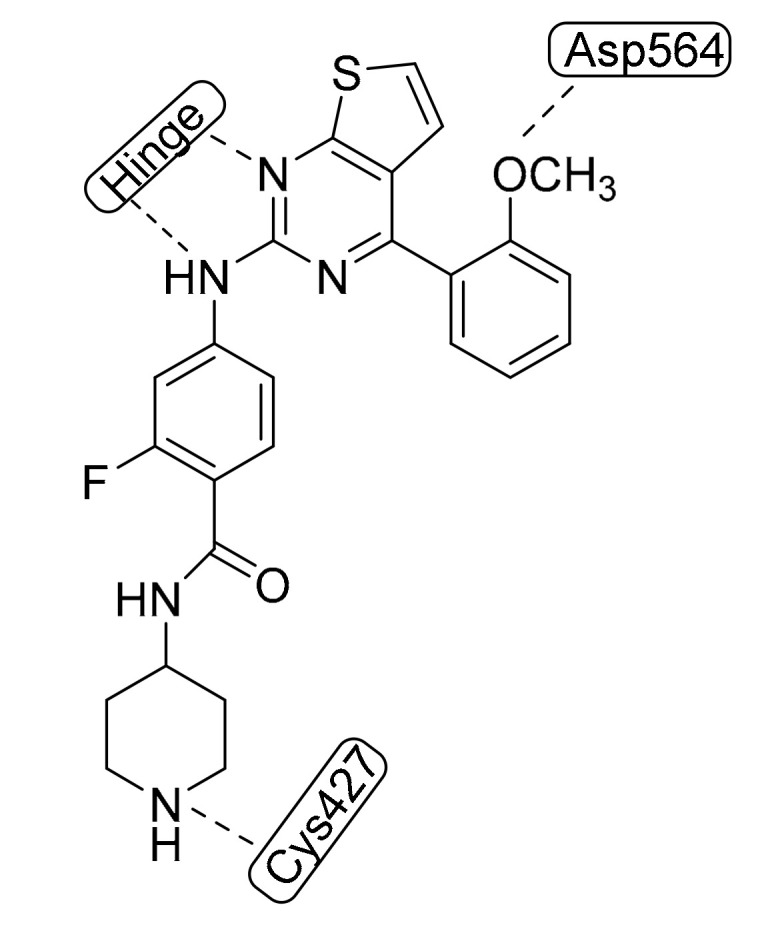
Chemical structure of the lead compound in the work by Zhao et al. [18].

Fak is a cytoplasmic protein tyrosine kinase overexpressed and activated in different solid cancers. It has been proposed as a potential target in cancer therapy, particularly in a metastatic phase. Zhao et al., miming the bioactive conformation of the well-known diaminopyrimidine motif and using a cyclization strategy, synthesized a large series of 7*H*-pyrrolo[2,3-*d*]pyrimidines as Fak inhibitors. Very recently, they obtained new 2,7-disubstituted-thieno[3,2-*d*]pyrimidine compounds by isosteric replacement of a pyrrole nucleus by a thiophene one [18], the most relevant being **1** (Figure 5), which showed an IC_50_ value of 28.2 nM in an enzymatic assay and displayed stronger potency than the reference compound TAE-226 in different biological assays.

As found in a docking study, compound **1** is anchored to the hinge region by double-dentate hydrogen bonds of pyrimidine nitrogen and aniline NH to the hinge region, whereas the *o*-methoxyl group is involved in the hydrogen bond with Asp564 of the DFG motif (Asp-Phe-Gly). Furthermore, the water-soluble “tail” of the piperidine moiety interacts with Cys427, confirming its important role in enzymatic inhibition. For all these reasons, this compound could be a promising lead for Fak-targeted anticancer drug discovery.

## 13. Tackling Coronas with EIDD-2801 Pills?

Highlighted by Katalin Prokai-Tatrai

The small molecule β-d-N^4^-hydroxycytidine (NHC) is a ribonucleoside analogue that inhibits viral RNA genome replication and is effective against a variety of viruses, including the cause of the current pandemic: coronavirus disease 2019 (COVID-19). NHC’s 5′-isopropyl ester prodrug, identified as EIDD-2801, was designed to improve pharmacokinetics and oral bioavailability in humans and nonhuman primates. It has recently been reported [19] that, when used as a prophylactic, oral administration of EIDD-2801 resulted in a significant viral load reduction and improved pulmonary function in mice infected with severe acute respiratory syndrome CoV (SARS-CoV) or Middle East respiratory syndrome CoV (MERS-CoV). Additionally, the weight loss of the experimental animals also markedly decreased. When given as a treatment within 12 or 24 h post-infection, the degree of lung damage could also be reduced. This window of therapeutic opportunity is expected to be significantly longer in humans. Overall, EIDD-2801 pills hold promise for not only the treatment of COVID-19 patients today but also against new CoVs that may emerge in the future.

## 14. Developing Efficient Mutant GTPase KRAS^G12C^ Inhibitors: Are We Ready for Mutant KRAS Yet?

Highlighted by Arun K. Sharma

KRAS, a key regulator of signaling pathways that cause cells to proliferate and differentiate, is a frequently mutated oncogene in human tumors, and G12C is the most common KRAS mutation in lung adenocarcinoma. Many efforts to develop KRAS^G12C^-specific inhibitors are currently underway—AMG510 (Amgen) and MRTX849 (Mirati/Array) are in clinical trials. Kettle et al. [20] further optimized covalent allosteric inhibitors of the mutant GTPase KRAS^G12C^ through conformational locking of a piperazine−quinazoline motif and linker modification and introducing a methyl group to the piperazine, leading to identification of a potent KRAS^G12C^ inhibitor with high selectivity, improved drug-like properties and excellent in-vivo efficacy. Although more studies would be required to establish the promise of this compound, this is a significant step forward in designing KRAS^G12C^ inhibitors. However, a recent report [21] suggests that resistance is inevitable for KRAS^G12C^ inhibitors since the cancer cells develop a heterogeneous response to the inhibitors and bypass inhibition by producing new active KRAS^G12C^ that does not bind the inhibitor, indicating that the challenge to develop efficient inhibitors and achieve complete responses in the clinic continues.

## 15. A Senolytic PROTAC Exploits the Restricted Expression Profile of CRBN to Lose Toxicity Associated with Its Parent Inhibitor

Highlighted by Matthieu Schapira

PROTACs are heterobifunctional molecules that recruit an E3 ligase to a target protein, leading to proteasomal degradation of the target. This novel modality is expected to extend the boundaries of the druggable genome [22]. He et al. have now established an additional benefit of PROTACs: the authors exploit the expression profile of the recruited E3 ligase to inhibit the target in tissues where it is pathogenic, while sparing it in tissues where it is beneficial [23].

Senolytics are compounds that selectively kill senescent cells implicated in age-related diseases such as osteoarthritis or cardiovascular and neurodegenerative diseases. ABT263, a protein interaction inhibitor that targets the anti-apoptotic proteins Bcl-xl and Bcl-2, is one of the most potent senolytics, but has unacceptable on-target toxicity, as Bcl-xl inhibition induces platelet apoptosis, resulting in severe thrombocytopenia. In an elegant work, He et al. have developed a PROTAC derived from ABT263 that recruits the E3 ligase CRBN to degrade Bcl-xl. Because CRBN is expressed in senescent cells but not in platelets, the PROTAC conserved the senolytic activity of ABT263 but lost its toxicity.

## 16. Possibility to Use Interleukin Itself for Treatment—IL-37 as an Example

Highlighted by Yasu-Taka Azuma

Up to now, more than 40 kinds of interleukins have been reported, and most of them induce inflammation. Therefore, until now, most of the therapeutic applications of interleukins have focused on blocking the action of interleukins using neutralizing antibodies. However, some interleukins, typically IL-10, have been found to have anti-inflammatory effects. Ballak et al. have reported that IL-37, which is an anti-inflammatory interleukin itself, has therapeutic potential [24]. They investigated the effectiveness of IL-37 treatment for improvement of physiological function in the elderly. Recombinant IL-37 treatment to old mice increased vascular endothelial function and endurance exercise capacity and improved whole-body insulin sensitivity and glucose tolerance. In addition, IL-37 has also been reported to show anti-inflammatory effects in patients with arteriosclerosis [25]. This interleukin therapy has further expanded the options for treating various diseases.

## 17. RNA-Based Nanoconstructs for Targeted Delivery of Paclitaxel to Breast Cancer: The Road to Drug Efficacy and Safety

Highlighted by Laura Cerchia

Targeted delivery of chemotherapy enhances the anticancer activity of the drug and limits side effects on healthy organs by increasing its concentration at the tumor site while dispensing lower absolute doses of the drug. Consequently, the development of novel cancer-targeted drug delivery systems represents one of the hottest areas of modern cancer research. Recently, Guo et al. [26] constructed a smart RNA-based multifunctional nanovector for targeted delivery of paclitaxel, whose use in the clinic is limited by scarce bioavailability and severe toxicity, to triple-negative breast cancer, by using aptamers for guaranteeing tumor targeting. Specifically, a ″X″-shaped RNA four-way junction nanostructure with ultra-thermodynamic stability was assembled with the extraordinary loading capability of twenty-four paclitaxel molecules as the prodrug. The intravenous injection of the construct, equipped with a previously validated and high efficacious anti-EGFR aptamer, in mice bearing orthotopic tumors greatly inhibited breast cancer growth. Further, nanoconstructs exhibited low/undetectable toxicity or immune responses in mice. Improving the transport of the drug to the tumor, by specific drug delivery platforms, will open up the possibility of accessing lower-cost and highly effective therapies.

## 18. New Insights into Mechanisms for Allosterically Modulating the Kappa Opioid Receptor with Nanobodies

Highlighted by Mariana Spetea

G-protein-coupled receptors (GPCRs) represent the most-targeted class of druggable proteins in the human genome [27]. The kappa opioid receptor (KOR) has emerged as an important therapeutic target for the treatment of pain and neuropsychiatric conditions. As a GPCR, KOR rapidly interconverts between multiple states, though the ability to visualize them is limited by a relative lack of suitable tools. Nanobodies (Nbs) provide a powerful platform to stabilize distinct receptor conformations. Che et al. [28] have demonstrated, via X-ray crystallography, a nanobody-targeted allosteric binding site of KOR, by which Nb6 stabilizes a ligand-dependent inactive state and Nb39 stabilizes an active-like state. They have shown how these two state-dependent nanobodies can provide real-time reporting of ligand stabilized states in cells in situ. Analysis of the KOR–Nb6 complex revealed a unique nanobody binding interface by which Nb6 negatively allosterically modulates KOR. The observation that this site can be transferred to other GPCRs to provide an engineered and functional allosteric site was also remarkable. Furthermore, given that Nb6 and Nb39 interact with distinct states of KOR, they can be used as biosensors to detect ligand-stabilized states in real time in living cells.

## 19. Is the Role of LMW Heparin in COVID-19 Infection Really Only Antithrombotic or Not?

Highlighted by Giangiacomo Torri

My answer to the question is no. It is well established that polyanionic molecules exhibit antiviral activities. Concerning heparin, the most relevant polyanionic macromolecule expressed by mammalian organisms, in the literature there are more than 3500 articles stating its antiviral activities with respect to different kinds of viruses. Among the 62 articles concerning SARS-CoV-2 and heparin, a basic research study by researchers experienced in glycosaminoglycans and proteins has demonstrated that a physical–chemical interaction between heparin and the viral protein occurs [29].

The current pandemic took us by surprise, and we found ourselves mostly unprepared to manage it in terms of experience and availability of adequate drugs, despite numerous studies on non-anticoagulant heparin derivative candidates. It is necessary to pursue a larger interchange between basic research, drug development and clinical knowledge. The goal is to minimize the dichotomy between clinical world and laboratory research.

## 20. Identification of Small Molecules Targeting Zika Virus NS2B-NS3 Protease via Fragment-Based Drug Discovery (FBDD) Approach

Highlighted by Simona Collina

In their recent publication, Quek et al. [30] have reported the successful application of a fragment-based drug discovery (FBDD) approach to identify small molecules targeting Zika virus (ZIKV) protease. ZIKV is a mosquito-borne flavivirus, which recently emerged as a global health threat. The viral NS2B-NS3 protease has an essential role in the maturation of viral proteins and represents an attractive therapeutic target for fighting ZIKV. The major drawback for development of NS2B-NS3 inhibitors is the negatively charged binding site. With the final aim to identify molecules with high affinity to the pocket and with drug-like properties, the authors adopted a FBDD approach. Employing a primary thermal shift assay, a fragment library of 1685 compounds was screened against the Zika protease, and twenty-two fragments were selected. After co-crystallization experiments, the X-ray structures of two complexes were successfully solved, showing that both compounds interact with the same site. These two hits were further characterized by STD NMR studies confirming their molecular interactions with the target. These fragments can be used as starting compounds for further growing into more potent inhibitors, thus paving the way for the discovery of novel effective ZIKV protease inhibitors.

## 21. Synthesis and Chemical Profile of New HIV-1 Capsid Targeting Peptidomimetic Analogous of PF74

Highlighted by Athina Geronikaki

The infection and dismantling of T cells engineered by the human immunodeficiency virus (HIV) induce human acute immunodeficiency syndrome (AIDS). The capsid protein (CA) involved in HIV-1 possesses a central role in various steps of the cycle of virus replication: in convening the core of functional capsid, regulating the kinetics of DNA entry and viral core uncoating with multiple host–factor interactions.

**Figure 6:**
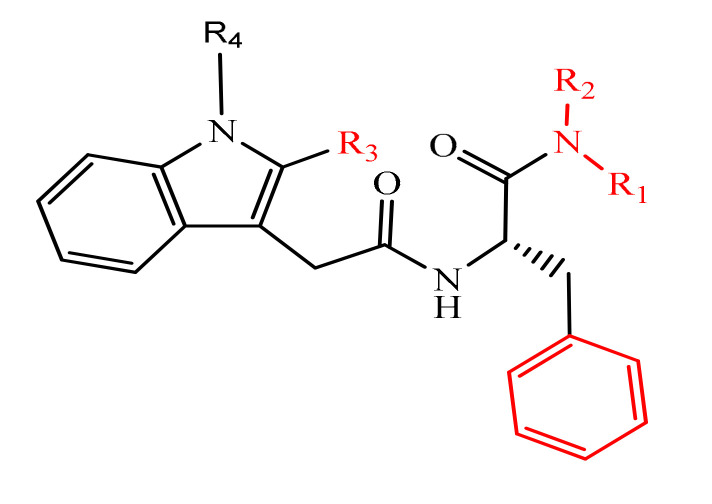
General structure of synthesized compounds.

Among all known small molecule chemotypes that target CA, the peptidomimetic PF74 has drawn particular interest since it exhibits a binding ability to the corresponding pocket. Wang et al. [31] have provided a thorough chemical optimization of PF74 by synthesizing and evaluating a broad amount of PF74 analog (Figure 6). Antiviral tests and CA hexamer stability accomplished on these analogs revealed occasional key pharmacophore aspects, including a phenyl moiety, a halogen *para*-substitution and a tiny alkyl group at the nitrogen in the aniline region, and the tolerance of carbon-3 and NH belonging to the indole skeleton for adjustment.

## 22. Let Cancer Cells Trip Themselves Up

Highlighted by Alfonso T. Garcia-Sosa

Many drug design efforts towards cancer therapy have concentrated on stopping the cellular processes of diseased cells. A new approach by Schulze et al. [32] overrides the spindle assembly checkpoint with small molecule inhibitors of MPS1 kinase, so that cancerous cells are allowed to progress to cell division with errors and mutations in their DNA in a so-called mitotic catastrophe. This has the effect of killing the cells at a later stage. Two separate chemical series were optimized with X-ray studies leading to two highly potent clinical candidates with favorable in-vivo PK properties: triazolopyridine BAY 1161909 and imidazopyridazine BAY 1217389. These ATP-competitive inhibitors make use of differently shaped binding pockets of MPS1 as well as different interactions with the protein. They are also synergistic in combination with the chemotherapy drug paclitaxel, which acts by interference with the normal function of microtubules during cell division. These compounds are the first MPS1 inhibitors to enter Phase I clinical trials, doing so with a novel mechanism of action.

## 23. Studies on SARSCoV-2 Spike Glycoprotein Open Doors for Covid-19 Drug Discovery

Highlighted by M. Helena Vasconcelos and Maria Emília Sousa

Three highly pathogenic zoonotic coronaviruses emerged recently: SARS-CoV in 2003, MERS-CoV in 2012, and SARS-CoV-2 in 2019. Coronavirus entry into host cells is mediated by the transmembrane spike (S) glycoprotein. In this study, Walls et al. [33] demonstrated that SARS-CoV-2 S uses ACE2 to enter cells. In addition, cryo-electron microscopy structures of the SARS-CoV-2 S glycoprotein revealed the presence of multiple conformational states of the SARS-CoV-2 S ectodomain trimer, providing important information for the discovery of vaccines and drugs to inhibit viral entry into cells. Finally, these authors observed a sequence and conformational conservation of the S protein fusion peptide regions between two coronaviruses (SARS-CoV-2 and SARS-CoV), putting forward the idea that infection by one of these two viruses could cause production of antibodies which might cross-react and neutralize both viruses as well as other related coronaviruses. Indeed, their results indicated that SARS-CoV S murine polyclonal antibodies potently inhibited SARS-CoV-2 S-mediated entry into cells.

## 24. With or Without ssDNA Genome, Pf4 Filamentous Phage Promotes the Stability of *P. Aeruginosa* Biofilm Formation and Consequently Increases Protection Against Antibiotics

Highlighted by Ivan Kosalec

Opposite to the well-known predation effects of bacteriophages on bacteria, filamentous phages express an interesting evolution-driven symbiotic relationship. Filamentous phages contain an ssDNA genome and very long and thin helically packed capsids. They do not harm the bacterial host, and, after egressing from the host, these phages symbiotically help bacteria to survive and to form more protected biofilms, especially during *Pseudomonas aeruginosa* infection [34,35]. The recent work of Tarafder et al. [36] has provided deeper insights into Pf4 filamentous phage and *P. aeruginosa* biofilm interactions, showing that Pf4 phages with ssDNA and capsid shell form protecting liquid shields around *P. aeruginosa* cells in biofilms. Removing the genome, Taragder et al. [36] found that phages with empty protein-structure capsid shells called “ghosts” formed liquid crystals around bacterial cells still protecting bacteria from antibiotic treatment. These observations have a great impact on bacteriophage–*P. aeruginosa* cell interactions in biofilms and reveal a more complex influence of Pf4 phages. Our strategy to modulate these effects on biofilms caused by *P. aeruginosa* must change as well.

## 25. An NMR Drug Screening Approach in Human Cells as a New Tool for Drug Potency Early Assessment

Highlighted by Tiziano Tuccinardi and Iola F. Duarte

A classical drug development workflow usually starts by screening the activity of multiple ligands against the selected target. Once a hit is identified, its chemical structure is used as a starting point for chemical modifications to improve potency and selectivity. However, promising candidates showing good performance in vitro often lack in-vivo activity, due to low membrane permeability or poor intracellular binding selectivity. Banci et al. reported an innovative experimental protocol to screen ligands in living human cells by “intracellular protein-observed” NMR spectroscopy, without requiring enzymatic activity measurements or other cellular assays. The method proposed is based on the observation and spectral deconvolution of ^1^H signals arising from free and ligand-bound fractions of the target macromolecule. Signal areas are used to build quantitative dose and time-dependent ligand binding curves, from which kinetic and thermodynamic parameters linked to cell membrane permeability and binding selectivity are retrieved [37]. Such an approach, applied in this work to carbonic anhydrase but extendable to other NMR-observable intracellular targets, represents a useful means to predict drug potency at an early stage of the development process.

## 26. Histone Deacetylase and Proteasome Dual Inhibitors for Overcoming Bortezomib Resistance

Highlighted by Jorge A. R. Salvador

Proteasome inhibitors (PIs), particularly bortezomib, have significantly improved the overall survival and quality of life of multiple myeloma (MM) patients. However, cancer resistance and relapse remain major difficulties in expanding the clinical utility of PI drugs.

**Figure 7:**
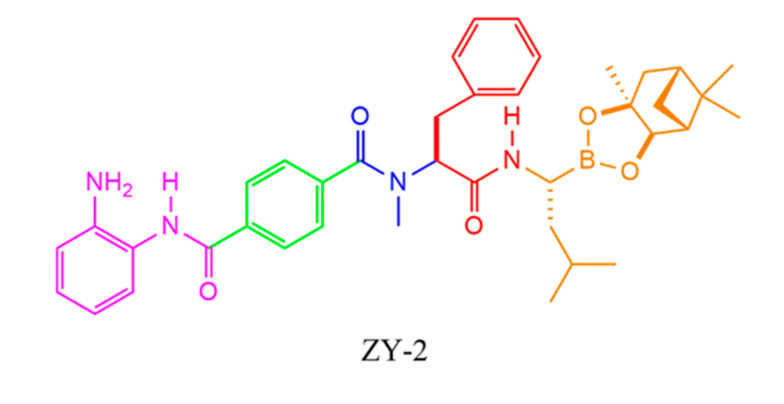
Chemical structure of ZY-2.

Hao Fang et al. [38] have designed and synthesized a novel series of peptide boronate derivatives as dual inhibitors targeting both histone deacetylases (HDACs) and proteasomes to address the resistance of bortezomib. The most potent inhibitors, ZY-2 (Figure 7) and ZY-13, showed excellent inhibition against proteasome and good selectivity against HDACs.

Specifically, ZY-2 also exhibited good antiproliferative activities on the MM cell lines RPMI-8226, U266, and KM3 (IC_50_ values of 6.66, 4.31, and 10.1 nM, respectively). In addition, ZY-2 showed more potent antiproliferative activities against the bortezomib-resistant MM cell line KM3/BTZ compared with bortezomib (IC_50_ values of 8.98 vs. 226 nM, *p* < 0.01). Moreover, ZY-2 also exhibited excellent potency in cell apoptosis induction and cell cycle arrest and decreased the levels of Sp1 and HDAC1 at low concentrations in the bortezomib-resistant MM cells.

## 27. BRAF-V600E Degraders: A New Weapon Against Melanoma

Highlighted by Massimo Bertinaria and Maurizio Pellecchia

The activating mutation V600E in the kinase BRAF is a major driver in several human cancers. BRAF-V600E produces a constitutively active kinase, which signals the expression of proliferation and survival genes in cancer cells [39]. BRAF-V600E inhibitors, such as vemurafenib, dabrafenib and encorafenib, are currently clinically approved for the treatment of BRAF-V600E-driven melanoma. One major drawback of these inhibitors, however, is represented by their paradoxical induced activation of wt-BRAF and concomitant activation of ERK signaling. Hence, in a potentially transformative new approach, Wang et al. have developed proteolysis-targeting chimeras (PROTACs) able to promote a selective proteasome-mediated degradation of mutant BRAF-V600E [40]. Two agents were obtained by linking vemurafenib to thalidomide using a short spacer, and by linking BI882370 (a newer and more selective BRAF-V600E inhibitor) to pomalidomide using a PEG-based spacer. The new agents are able to induce degradation of BRAF-V600E at nanomolar concentrations and inhibit the growth and viability of A375 melanoma cells. Most importantly, the agents do not promote degradation of wild-type BRAF, hence sparing non-neoplastic human cells.

## 28. Target-Guided Synthesis of Noncanonical DNA-Binding Transcriptional Modulators

Highlighted by Jussara Amato

G-quadruplex and i-motif are four-stranded noncanonical nucleic acid structural arrangements. Recent evidence suggests that these DNA structures exist in living cells and are involved in several cancer-related processes, like the transcriptional regulation of proto-oncogenes, thus representing an attractive target for anticancer drug discovery. Considering the dynamic nature of DNA structures, the development of small molecules that can bind to them with high affinity and selectivity has always been challenging and time consuming. Dash et al. have reported an innovative example of a target-guided synthetic approach, using DNA-immobilized magnetic nanotemplates and an array of azide and alkyne fragments, in which the active site of the biological target controls the assembly of the best binding fragments [41]. The in-situ-generated lead compounds can be easily isolated by simple magnetic decantation, and DNA templates can be recovered and recycled. The identified small molecules effectively showed high affinity and specificity for the target, as well as the ability to modulate gene expression. This approach might pave the way to in-situ development of transcriptional modulators for therapeutic interventions.

## 29. The Crystal Structure of the SARS-CoV-2 Main Protease: New Hopes to Fight a Threatening Disease

Highlighted by Giulio Rastelli

**Figure 8:**
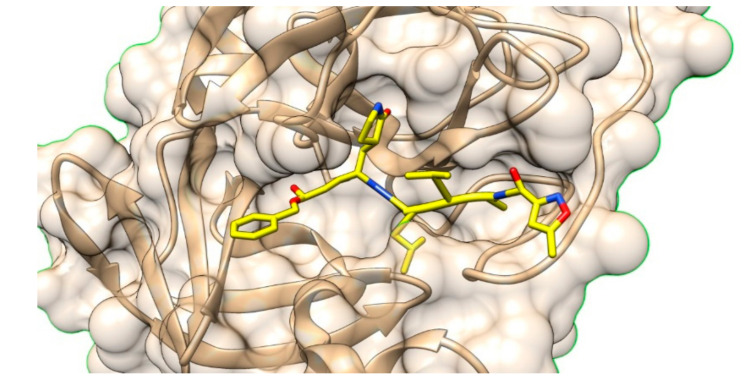
Crystal structure of the SARS-CoV-2 main protease in complex with ligand N3 (PDB code 7BQY).

The severe acute respiratory syndrome coronavirus 2 (SARS-CoV-2), the new virus responsible for the COVID-19 outbreak, is posing a real threat to global health. Jin et al. [42] have described the crystal structure of the main protease (Mpro) of SARS-CoV-2 (Figure 8), an essential enzyme of the viral life cycle. The authors firstly developed a fluorescence resonance energy transfer assay compatible with high-throughput screening, then solved the crystal structure of SARS-CoV-2 Mpro in complex with the Michael acceptor inhibitor N3 at 2.1 Å and 1.7 Å resolutions (PDB codes 6LU7 and 7BQY, respectively). The ligand binds in the substrate cleft by adopting an extended conformation (Figure 8); it is covalently bound to catalytic cysteine and establishes several hydrogen bonds and hydrophobic contacts. Remarkably, high-throughput screening of a library of about 10,000 compounds identified six ligands (disulfiram, carmofur, ebselen, shikonin, tideglusib and PX-12) able to inhibit the SARS-CoV-2 protease with micromolar IC_50_, with ebselen and N3 also showing antiviral activity.

Without doubt, the availability of this crystal structure will greatly support the rational identification of drug leads and/or repurposed drugs for the treatment of COVID-19, for which effective therapeutics are still lacking.

## 30. Stalobacin I: The Newest Member of the Remarkable Family of Peptide-Based Antibiotics

Highlighted by Paula A. C. Gomes

Resistance to antibiotics currently available in clinics is reaching alarming levels, and can affect anyone, of any age, gender or socio-economic status, in any region of the world. Of particular concern are the so-called ESKAPE pathogens, multi-drug resistant (MDR) strains of *Enterococcus faecium*, *Staphylococcus aureus*, *Klebsiella pneumoniae*, *Acinetobacter baumannii*, *Pseudomonas aeruginosa*, and *Enterobacter* bacteria that are major players in nosocomial infections. When current antibiotics fail to mitigate severe and life-threatening infections, “last-resort” options come into play, including use of the well-known natural lipocyclopeptides daptomycin and colistin, or the more recent synthetic glycopeptide telavancin [43]. This prominent role of peptide-based antibiotics in the battle against MDR infections has been consolidated over recent years by the discovery of other natural peptides with non-canonical structures, like teixobactin [44] or lungdunin [45]. Stalobacin I, a lipopeptide now identified by Matsui et al., comes to enrich the portfolio of peptide-based antibiotics with potent action against MDR Gram-positive bacteria [46]. Hopefully, similar success will soon emerge regarding discovery of as-yet-elusive alternatives to polymyxins or colistin as peptide-based antibiotics to fight MDR Gram-negative bacteria.

## 31. Are We Now One Step Closer to the Discovery of new Antibiotics or Overcoming Their Resistance with Artificial Intelligence?

Highlighted by Rita C. Guedes and Jean-Marc Sabatier

Antibiotic-resistant bacteria have risen sharply and are a major challenge in drug discovery. To address this challenge, Collins et al. [47] have applied a deep learning approach based on neural network training to identify new chemotypes with putative antibacterial activity. Their combined approach to identify potential lead compounds consisted of the training of a deep neural network model to predict growth inhibition of *E. coli* (2335 molecules), which was then applied to the screen of multiple chemical libraries (>107 million molecules). Selected compounds (the best ranked as structurally “unique” compared with other antibiotics and devoid of apparent toxicity) were then tested against bacterial growth. Among other potential compounds, the c-Jun N-terminal kinase inhibitor SU3327 (halicin) was identified as a potent *E. coli* growth inhibitor. This work proves the importance of the use of machine learning in our drug discovery pipelines to expand conventional chemical space.

## 32. Design and Synthesis of Oral Bioavailable Chromone Derivatives as Novel Selective BRD4 Inhibitors

Highlighted by Ana Estévez-Braun

**Figure 9:**
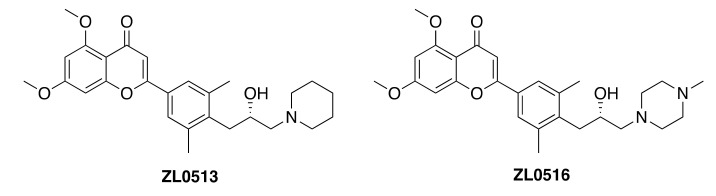
Structures of ZL0513 and ZL0516.

BET family proteins, especially BRD4, represent a promising epigenetic target for human diseases, such as cancers, inflammations, CNS disorders, heart failure, and HIV infection. A series of compounds based on the chromone core were designed and obtained through a scaffold hopping approach. Two of them, ZL0513 and ZL0516 (Figure 9), displayed nanomolar binding affinity (IC_50_ 67–84 nM) with BRD4 BD1 [48]. Their binding modes were validated by the cocrystal structure of ZL0516 in complex with human BRD4 BD1. These compounds also showed significant in-vivo efficacy in a murine model of TLR3-induced acute airway inflammation. Finally, pharmacokinetic studies revealed that both compounds are orally available, indicating their potential as therapeutic agents in inflammatory diseases.

## 33. Aptamers as a Therapeutic Tool Against Prion Diseases

Highlighted by Bruno Pagano

Prion diseases, also known as transmissible spongiform encephalopathies, are fatal neurodegenerative disorders affecting humans and animals. An innovative and promising therapeutic approach is based on the use of nucleic acid aptamers to block the hallmark event in the prion diseases, i.e., the conversion of prion protein into an abnormal form. Starting from a previously identified RNA aptamer, Mashima et al. have rationally developed a new aptamer that exhibited very high anti-prion activity [49]. Rationalization of the high anti-prion activity was also provided on the basis of RNA structure determination. The achievement of an IC_50_ value of 100 nM represents a substantial advance that has opened the realistic possibility of applying these nucleic acid molecules for therapeutic use. The development of drugs against prion diseases is long overdue. These results may pave the way to the development of new, powerful tools against diseases lacking effective therapy.

## 34. A Newly Designed γ–Lactam-Siderophore Conjugate Shows Significant Antibiotic Activity Against Multidrug-Resistant Gram-Negative Bacteria

Highlighted by Stefano Mangani

**Figure 10:**
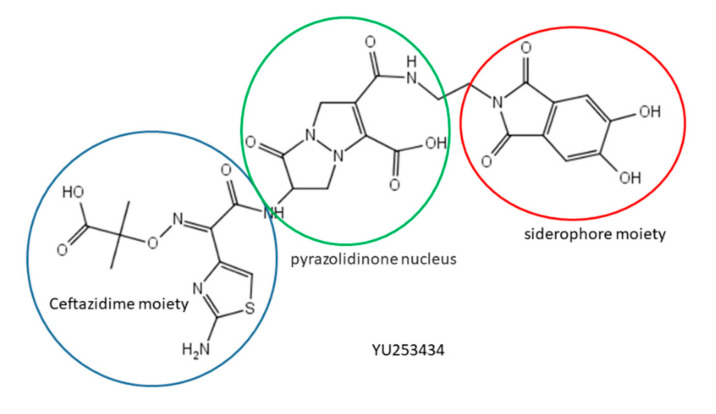
Structure of YU253434.

The concept of using the siderophore importers present in Gram-negative bacteria to facilitate the intake of antibiotics into the periplasmic space [50] has been applied to the development of a new compound endowed with interesting activity against multidrug-resistant (MDR) bacteria. Goldberg et al. [51] have reported the design, synthesis, structural characterization and antimicrobial activity of a novel pyrazolidinone antibiotic containing a dihydroxyphthalimide siderophore mimetic. The compound, dubbed YU253434, contains the 2-(2-aminothiazol-4-yl)-2-(2-carboxypropan-2-yloxyimino)acetamido moiety of ceftazidime linked to the pyrazolidinone nucleus, which in turn bears a dihydroxyphtalimide acting as a siderophore mimic (Figure 10). YU253434 acts a covalent inhibitor of PBPs (Penicillin Binding Proteins) by binding to the catalytic serine residue as shown by the X-ray crystal structure of its complex with PBP3.

YU253434 is stable with respect to β-lactamases of all classes. Its MICs against clinical isolates of relevant MDR bacteria compare favorably with those of β-lactam antibiotics used in clinics. The structure of the YU253434 bound to PBP3 provides hints for further optimization of this promising compound.

## 35. Tackling SARS-CoV-2: Key Hints for Structure-Based Design of Broad-Spectrum Inhibitors of 3CL Protease

Highlighted by Rino Ragno, George Kokotos, Margherita Brindisi and Florenci V. González

**Figure 11:**
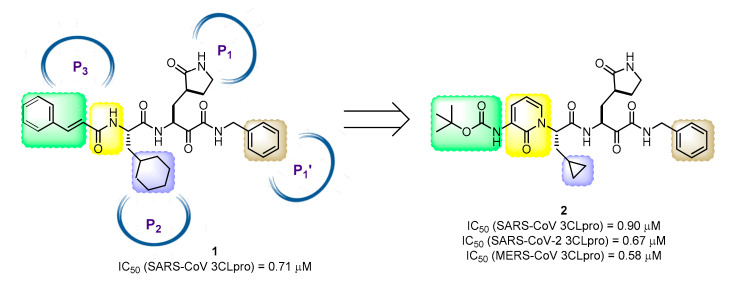
Structure of α-ketoamide-based broad-spectrum 3CL^pro^ inhibitors **1** and **2**.

The COVID-19 pandemic caused by SARS-CoV-2 has generated an unprecedented health emergency worldwide. The development of broad-spectrum antivirals useful for future outbreaks of pathogenic coronaviruses is of absolute priority. Proteolytic enzymes, essential for producing mature virions, represent attractive targets for drug development. One of them is the SARS-CoV-2 chymotrypsin-like protease (3CL^pro^), a protease utilizing a Cys–His catalytic dyad for its hydrolytic activity. In their quest for novel broad-spectrum 3CL^pro^ inhibitors (Figure 11), Hilgenfeld et al. identified compound **1**, featuring an α-ketoamide warhead [52]. Based on **1**, structure modification led to new compounds with an extended activity spectrum against coronaviruses, while improving the drug-like profile [53]. In order to enhance metabolic stability and decrease plasma protein binding, the P3-P2 amide was embedded into a pyridone ring, and the terminal cinnamoyl portion was replaced by a *tert*-butoxycarbonyl group. The X-ray structures of the unliganded SARS-CoV-2 3CL^pro^ and its complex with an α-ketoamide inhibitor were solved and enabled structure-based drug design. Compound **2** effectively inhibited the main coronaviruses 3CL^pro^ (SARS-CoV-2, SARS-CoV and MERS-CoV) with an average IC_50_ of 0.79 μM and exhibited promising antiviral potency in cell-based assays, with associated improved half-life and favorable lung tropism.

## 36. Drug Discovery and Central Nervous System Diseases: Neuroinflammation as a Step Forward

Highlighted by Fernanda Borges

Central nervous system (CNS) diseases are a large group of neurological disorders with heterogeneous clinical and pathological expressions. The intricate network of regulatory mechanisms remains largely elusive, and, to date, no specific factor has been identified as a direct cause. Furthermore, the existing drugs in therapy are only palliative and fail to modify disease progression.

Identifying and validating novel and druggable CNS targets remains a hot topic in drug discovery, in particular those involved in neuroinflammation. Interestingly, the prostanoid EP2 receptor has been associated with neuroinflammation and neurodegeneration in CNS diseases. In this context, the work of Thota Ganesh’s research team reports the discovery of new EP2 antagonists, obtained after a lead optimization process. This work describes the improvement of selectivity and drug-like properties of the brain-permeating lead TG6-10-1. The optimized antagonist, 2-[(4,6-dimethylpyridin-2-yl)amino]-*N*-[2-(2-methyl-1*H*-indol-3-yl)ethyl]pyrimidine-5-carboxamide hydrochloride (TG11-77·HCl), has a higher selectivity index, suitable brain permeability and excellent water solubility [54].

## 37. Evaluation of Nonvaccination Preventive Mechanisms to Fight Antibiotic Resistence: Evidence from Enteropathogenic *E. coli* Infection

Highlighted by Mariarosaria Miloso

Antibiotic resistance is a crucial problem for human health, and it is fundamental to explore alternative therapeutic options to treat bacterial infections. Qiu et al., by in-vitro and in-vivo experiments, demonstrated the effectiveness of a specific reactive peptide to block enteropathogenic *E. coli* (EPEC) infection [55]. EPEC infection starts with the attachement of a bacterium to the surface of an intestinal cell in order to inject Tir protein into the cytoplasm. Tir interacts with Nck adaptor, which, in turn, allows the interaction of Tir with the Arp2/3 complex involved in actine reorganization and polimerization. Formation of an actin pedestal is responsible for cell lesions and specific symptoms of EPEC infection. Nck is an adaptor with different domains, among which the second SH3 domain, Nck-SH3.2, is specifically involved in Tir interaction. Qui et al. [55] have reported the design of a synthetic peptide that binds to the Nck-SH3.2 domain at the level of Cys48. This interaction blocks the Nck binding site for Tir. Consequently, Tir cannot interact with Arp2/3 complex and no actin reorganization occurs blocking EPEC infection. The reactive peptide, which acts on cells rather than on the pathogen, may be a promising approach to fighting drug resistance.

## 38. Glucuronide Prodrug, PEGylation, Albumin Binder, Self-Immolative Linker, and a Potent Toxin: An Ideal Ensemble for Efficient Tumor Suppression Upon Highly Localized Bioactivation

Highlighted by Jarkko Rautio and Diego Muñoz-Torrero

**Figure 12:**
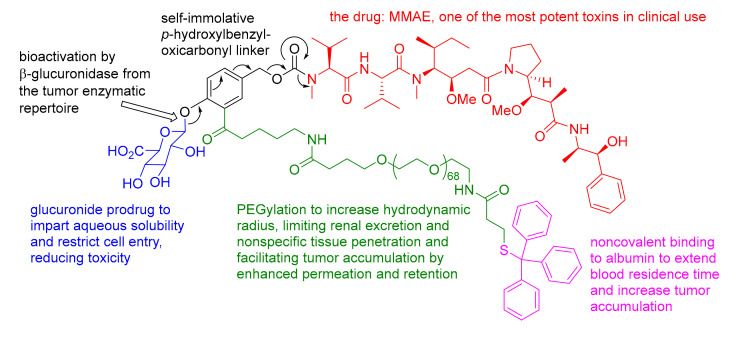
Chemical structure and mechanism of the bioactivation of the Tr-PEG glucuronide supramolecular MMAE prodrug.

Zelikin’s group [56] have reported efficient suppression of tumor growth by enzyme prodrug therapy, which takes advantage of noncovalent albumin binding and, subsequently, delivery of the prodrug to the tumor as well as the endogenous enzymatic repertoire of the tumor. To this end, a total of eight glucuronide prodrugs of highly cytotoxic monomethyl auristatin E (MMAE) were engineered, where a self-immolative scaffold based on *o*-substituted *p*-hydroxybenzyl alcohol provided a spacer between the drug, glucuronide, and either the molecular (e.g., alkyne), macromolecular (e.g., PEGs), or supramolecular (e.g., trityl (Tr)-PEG) protraction arm. While most prodrugs significantly masked the toxicity of MMAE and were well tolerated after subcutaneous administration to an MDA-MB-231 ectopic xenograft mouse model, only the Tr-PEG glucuronide prodrug (Figure 12) afforded statistically significant suppression of tumor growth. The best prodrug could not be predicted from the in-vitro toxicity screens or in-vivo derived pharmacokinetics parameters, but favorable anticancer effects correlated only with accumulation of the released payload from the prodrug at the tumor site. This is a very useful approach for all modalities of cancer detection, imaging, and treatment relying on enhanced tumor localization!
